# Melanocortin 4 Receptor-Dependent Mechanism of ACTH in Preventing Anxiety-Like Behaviors and Normalizing Astrocyte Proteins after Early Life Seizures

**DOI:** 10.1523/ENEURO.0564-24.2025

**Published:** 2025-03-07

**Authors:** Mohamed R. Khalife, Colin Villarin, Juan Manuel Ruiz, Sam A. McClelland, Khalil Abed Rabbo, J. Matthew Mahoney, Rod C. Scott, Amanda E. Hernan

**Affiliations:** ^1^Division of Neuroscience, Nemours Children’s Health, Wilmington, Delaware 19803; ^2^Psychological and Brain Sciences, University of Delaware, Newark, Delaware 19716; ^3^Perelman School of Medicine, University of Pennsylvania, Philadelphia, Pennsylvania 19104; ^4^Bowdoin College, Brunswick, Maine 04011; ^5^University of Vermont, Burlington, Vermont 05401; ^6^The Jackson Laboratory, Bar Harbor, Maine 04609; ^7^Neurosciences Unit, Institute of Child Health, University College London, London WC1N 1EH, United Kingdom

**Keywords:** ACTH, anxiety, astrocytes, early-life seizures, epilepsy, melanocortin 4 receptor

## Abstract

Epilepsy, affecting millions globally, often leads to significant cognitive and psychiatric comorbidities, particularly in children. Anxiety and depression are particularly prevalent, with roughly a quarter of pediatric epilepsy patients having a comorbid diagnosis. Current treatments inadequately address these issues. Adrenocorticotropic hormone (ACTH), a melanocortin peptide, has shown promise in mitigating deficits after early-life seizures (ELS), potentially through mechanisms beyond its canonical action on the melanocortin 2 receptor. This study explores the hypothesis that recurrent ELS is associated with long-term anxiety and that treatment with ACTH can prevent this anxiety through a mechanism that involves the melanocortin 4 receptor (MC4R) in the brain. Our findings reveal that ACTH ameliorates anxiety-like behavior associated with ELS, without altering seizure parameters, in wild-type but not in male and female MC4R knock-out mice. Our findings also show that knocking-in MC4R in either neurons or astrocytes was able to rescue the anxiety-like behavior after ACTH treatment. Furthermore, our results show that ACTH normalizes important astrocytic proteins like glial fibrillary acidic protein and aquaporin-4 after ELS. This suggests that ACTH's beneficial effects on anxiety are mediated through MC4R activation in both neuronal and astrocytic populations. This study underscores the therapeutic potential of targeting MC4R as a treatment, highlighting its role in mitigating anxiety-like behaviors associated with ELS.

## Significance Statement

This study reveals a novel mechanism by which ACTH mitigates anxiety-like behaviors and normalizes key astrocyte markers, including glial fibrillary acidic protein and aquaporin-4, following early-life seizures (ELS) in a melanocortin 4 receptor (MC4R)-dependent manner. This challenges the notion that ACTH's primary effects are mediated through the melanocortin 2 receptor in the adrenal cortex. This study further shows that ACTH's effects extend beyond seizure control, targeting psychiatric comorbidities, challenging the prevailing assumption that comorbidities are a result of seizures. These findings not only expand our understanding of ACTH's beneficial effects through MC4R in both neuronal and astrocyte populations but also suggest new avenues for treating ELS-related comorbidities.

## Introduction

Epilepsy, affecting 50–65 million people worldwide, is a neurological condition characterized by an enduring predisposition to generate seizures with significant neurobiological, cognitive, and social implications (Fisher et al., 2014; Beghi, 2020). Approximately 30–40% of children with epilepsy experience cognitive, psychiatric, or behavioral comorbidities, particularly anxiety disorders (Ettinger et al., 1998; Ekinci et al., 2009; LaGrant et al., 2020), significantly impacting quality of life (Lodhi and Agrawal, 2012; Reilly et al., 2014; Ahmed et al., 2022; Sayed et al., 2023). Current antiseizure therapies fail to adequately address these comorbidities (Lodhi and Agrawal, 2012; Valeta, 2017; Khalife et al., 2022).

ACTH, a melanocortin family peptide within the hypothalamus–pituitary–adrenal axis, has been utilized as a treatment for decades in childhood epilepsies, particularly infantile spasms (Charuvanij et al., 1992; Kivity et al., 2004; Okumura et al., 2006; Darke et al., 2010). The canonical mechanism of ACTH involves activating the melanocortin 2 receptor (MC2R) in the adrenal glands, playing a role in steroidogenesis (Roy et al., 2007; Novoselova et al., 2019). However, because ACTH agonizes all melanocortin receptors (MCRs), not just the canonical MC2R, we demonstrated that rodents with a history of early-life seizures (ELS) treated with ACTH, but not a corticosteroid, showed significant improvements in fear extinction learning and attention (Hernan et al., 2014; Massey et al., 2016), as well as normalized gene expression in the brain related to synaptic plasticity and cell communication, without altering seizure parameters (Brabec et al., 2022). Taken together, these data support a role for ACTH acting at an additional pathway, with actions above and beyond canonical corticosteroid release.

The melanocortin receptors, MCRs1–5, exhibit distinct tissue distributions and functions (Yang, 2011; Cai and Hruby, 2016). Activation of melanocortin 4 receptor (MC4R), primarily expressed in the CNS, has been shown to be neuroprotective in neurodegenerative diseases (Yang et al., 2000; Farooqi et al., 2003; Daniels et al., 2009). Recent studies indicate that MC4R activation can decrease cell death, enhance cognitive performance, and regulate synaptic plasticity in the hippocampus (HC; Yang et al., 2000; Farooqi et al., 2003; Daniels et al., 2009; Spaccapelo et al., 2011; Shen et al., 2013, 2016; Giuliani et al., 2014). For instance, activating the hippocampal MC4R circuit alleviated synaptic plasticity impairments in Alzheimer's disease (Shen et al., 2016) indicating that MC4R has therapeutic potential in conditions where synaptic function is compromised.

Research has mainly focused on neuronal function after ELS, and it was shown that ELS can disrupt the hippocampal–prefrontal cortex network and alter synaptic plasticity leading to cognitive and psychiatric deficits. However, there is a significant gap in the understanding of the role of astrocytes, which play a vital role in the brain's response to injury, after ELS. Activation of MC4R in astrocytes promotes anti-inflammatory effects and modulates cell survival proteins (Caruso et al., 2007). Astrocytes are crucial in epilepsy, resetting ion balance and recycling neurotransmitters after seizure (Vezzani et al., 2022). Astrocyte alterations contribute significantly to seizure predisposition (Binder and Steinhäuser, 2021). For instance, increased levels of glial fibrillary acidic protein (GFAP) and altered aquaporin-4 (AQP4) expression are both linked to increased seizure susceptibility and worsened seizure activity (Heuser et al., 2010; Hubbard et al., 2016; Szu et al., 2020; Lu et al., 2021; Vezzani et al., 2022; Sumadewi et al., 2024). However, the role of MC4R activation in mitigating astrocyte dysfunction in epilepsy has not been explored.

We have previously shown in two rat models that ACTH can improve cognitive outcomes after ELS and that ACTH treatment in control rats did not alter cognitive function (Hernan et al., 2014; Massey et al., 2016). Here, we show for the first time that ELS is associated with significant anxiety in a mouse model, which is ameliorated by ACTH. This study tested whether ACTH exerts its beneficial effects through MC4R in both neuronal and astrocyte populations. Our findings underscore a crucial role for MC4R in ACTH's positive effects and its potential to alleviate anxiety-like behavior.

## Materials and Methods

### Animals

Male and female mice were used in all studies. C57BL/6J (strain #000664), MC4r KO (B6.129S4-Mc4rtm1Lowl/J, strain #032518), Syn1-cre (B6.Cg-Tg(Syn1-cre)671Jxm/J, strain #003966), and Gfap-cre (B6.Cg-Tg(Gfap-cre)77.6Mvs/2J, strain #024098) mice from The Jackson Laboratory were used in the experiments. We used littermates and equivalently bred mice (WTs, KOs, and KIs from the same corresponding litters and wild-type, knock-in, and knock-out animals bred in parallel whenever possible) to reduce confounding genetic variability. All mice were housed in the same animal room and provided with food and water *ad libitum* and maintained on a 12 h light/dark cycle with consistent temperature and humidity, identical bedding, and enrichment. Each cage contained 3–5 mice of the same sex and treatment group to avoid social stress. The same experimenter provided regular husbandry and was the sole handler for all behavioral assays. This approach minimized discrepancies in handling or environment that could inadvertently influence anxiety measures. All experiments were performed in accordance with the guidelines instated by the National Institutes of Health’s “Guide for the Care and Use of Animals.” The animal protocols were approved by the Institutional Animal Care and Use Committee at Nemours Children's Health.

MC4R knock-out mice were maintained as homozygous, and littermates with both copies of the receptor were obtained from The Jackson Laboratory and used as controls. This strain has a floxed stop codon in front of the MC4R gene, allowing us to knock the receptor back using Cre recombinase. For the knock-in (KI) experiments, we used a breeding strategy with homozygous MC4R KO mice and hemizygous GFAP-Cre or Syn1-Cre mice to reexpress MC4R in astrocytes or neurons, respectively. Multiple lines of evidence have confirmed the astrocyte-specific expression of GFAP-Cre (B6.Cg-Tg(Gfap-cre)77.6Mvs/2J) through strong colocalization of Cre with GFAP+ astrocyte immunoreactivity in various brain regions (Jia et al., 2020, 2021; Itoh et al., 2023), while Syn1-Cre (B6.Cg-Tg(Syn1-cre)671Jxm/J) has been validated as neuron-specific (Zhu et al., 2001; Zheng et al., 2013), both with no off-target effects. We verified the presence of the Cre transgene by polymerase chain reaction genotyping. Male hemizygous GFAP-Cre, male hemizygous Syn1-Cre, and female homozygous KO mice were used. We bred homozygous MC4R KO females with hemizygous GFAP-Cre males in the first cross. We genotyped the F1 generation and selected heterozygous KO mice that were also Cre positive. The one floxed allele of the MC4R gene was reintroduced by the Cre recombinase, resulting in heterozygous MC4R knock-out mice. F1 generation heterozygous KO/Cre+ mice were bred with homozygous MC4R KO mice. Seventy-five percent of the offspring from breeding a heterozygous KO/Cre+ mouse with a homozygous KO mouse will be Cre positive. The Syn1-Cre mice were bred in a similar manner.

#### Handling and habituation

All animals were handled daily by a single experienced experimenter for routine husbandry and any procedural injections. The same experimenter conducted all behavior tests, minimizing variability due to multiple handlers. Mice were transferred to the testing room at least 60 min prior to the start of any behavioral task for habituation. During testing, the experimenter remained outside the room to reduce potential stress or distraction. This consistent handling and habituation protocol was designed to minimize anxiety and thigmotaxis, enhancing the validity of locomotion, exploration, and anxiety measures.

#### Early-life seizure mouse model

Seizures begin on postnatal day 10 (p10). Twenty total seizures were administered for 5 d from p10 to p14. Wild-type and knock-out mice were divided into three groups each: WT control vehicle-treated, vehicle-treated ELS, and ACTH-treated ELS and KO control vehicle-treated, vehicle-treated ELS, and ACTH-treated ELS (WT: *N* = 7 control vehicle-treated, *N* = 8 vehicle-treated ELS, *N* = 9 ACTH-treated ELS; KO: *N* = 7 control vehicle-treated, *N* = 7 vehicle-treated ELS, *N* = 6 ACTH-treated ELS). Knock-in mice were split into two groups each (*N* = 6 MC4R Syn1 KI vehicle-treated ELS, *N* = 8 MC4R Syn1 KI ACTH-treated ELS, *N* = 5 MC4R GFAP KI vehicle-treated ELS, *N* = 5 MC4R GFAP KI ACTH-treated ELS; Table 1). An hour before every seizure, mice received subcutaneous injections of their respective drug or vehicle. Animals took a 1 h break between each of their four daily seizure sessions. Four-animal groups were housed in individual sections of a custom-designed and built plexiglass chamber, which were linked to a central chamber that contained flurothyl. All sections of the chamber have equal access to the central chamber containing the flurothyl when the section doors are closed, but a sliding plastic panel cuts off flurothyl access once the section is opened for quick evacuation of the flurothyl.

**Table 1.** Wild-type, knock-out, and knock-in mice numbers

Animal group	Number of animals
WT vehicle-treated control	7
WT vehicle-treated ELS	8
WT ACTH-treated ELS	9
KO vehicle-treated control	7
KO vehicle-treated ELS	7
KO ACTH-treated ELS	6
MC4R Syn1 KI vehicle-treated ELS	6
MC4R Syn1 KI ACTH-treated ELS	8
MC4R GFAP KI vehicle-treated ELS	5
MC4R GFAP KI ACTH-treated ELS	5

### Flurothyl induction

A flurothyl dose of 0.02 ml (Sigma-Aldrich) was dispensed into the central chamber on a filter paper and allowed to diffuse into the connected chambers containing individually placed animals. At incremental doses of 0.01 or 0.02 ml, flurothyl was given to elicit seizures with a minimum interval of 1 min between each administration. The animal's chamber was immediately evacuated upon the onset of a tonic–clonic seizure. Seizure sessions were recorded through webcams for close monitoring and further offline analysis. Animal-specific metrics, such as latency to seizure and duration of seizure, were documented by an observer and confirmed through video analysis.

### Drug administration

Mice in the ACTH group received subcutaneous injections with an ACTH in 5% gelatin solution at a dose of 150 IU/m^2^, diluted with 5% gelatin to a total volume of 0.1 ml. Vehicle control and vehicle ELS littermates received 0.1 ml subcutaneous injections of the same solution vehicle as their littermates. All mice received drug administration once daily, 1 h prior to each day's seizure inductions.

### Seizure latency and duration

To evaluate the differences in seizure latency and duration between various experimental groups, we conducted a time-to-event analysis using the Cox proportional hazards model. We employed a shared frailty Cox proportional hazards model due to its robustness in handling time-to-event data, allowing us to assess the effect of multiple covariates on the hazard of seizure occurrence adjusting for multiple measurements per animal. To account for day-specific effects, we stratified the Cox model by seizure day. This stratification ensures that our results are adjusted for any variations in seizure risk that may occur from day to day. This method enabled us to compare the seizure characteristics across different treatment and genotype groups, providing insights into the effects of various interventions on seizure dynamics.

### Immunohistochemistry

Forty-eight hours after the last seizure, animals were perfused followed by brain collection. Brains were fixed with 4% paraformaldehyde (PFA) for 4 h and then transferred into a 20% sucrose solution for cryopreservation. Brains were sliced at 40 um for the prefrontal cortex (PFC) and hippocampus (HC) and fixed on charged slides for staining. A 2 d immunohistochemistry protocol was used to assess the acute effects of ELS and ACTH treatment on astrocyte protein expression. Day 1 consisted of two 15 min washes in 1× PBS, 1 h of blocking and tissue permeabilization, and an overnight primary antibody incubation at 4°C. Day 2 consisted of three 15 min washes in 1× PBS, one 2 h secondary antibody incubation in the dark at room temperature, three additional 15 min washes in 1× PBS, coverslip mounting using Fluoroshield with DAPI, and coverslip sealing. Blocking buffer was composed of 10% normal goat serum and 0.5% Triton X-100 in 1× PBS. Tissues were costained with primary antibody, glial fibrillary acidic protein (GFAP; 1:1,000; ab4674, Abcam), and the conjugated secondary antibody, Alexa Fluor 555 (ab150170, Abcam), alongside another astrocytic biomarker aquaporin 4 (AQP4; 1:500; NBP1-87679, Novus Biologicals) and the secondary antibody, Alexa Fluor 488 (1:1,000; ab150077, Abcam). Stained sections were imaged at 40× using confocal microscopy, processing *Z*-stacks into maximum-intensity projections to visualize all tissue depths of fluorescent regions of interest (ROIs) and image analysis using ImageJ and the Colocalization Finder plug-in.

### Open field task

At p50, mice were placed in a 46 cm × 46 cm arena in an isolated room and allowed to explore freely for 10 min. Sessions were video recorded and analyzed via ANY-maze for total locomotion, center entries, and relative time spent in the center in order to obtain measures for spontaneous activity and exploration.

### Light/dark box task

At p50, mice were placed in a 46 cm × 27 cm × 30 cm acrylic box. One-third of the box was a dark area, while two-thirds of the box was exposed to light. A plexiglass door allowed the animals to enter either compartment of the box. The mouse was placed in the light compartment of the box and allowed to freely explore for 10 min. Animals were video recorded and analyzed via ANY-maze for time spent in each compartment in order to test anxiety-like behavior.

### Software validation

The open field and light/dark box sessions were recorded and analyzed using ANY-maze (Stoelting), an automated video tracking software widely used for rodent behavior assessments (Seibenhener and Wooten, 2015). ANY-maze offers accurate real-time tracking of each animal's position, speed, and zone entries, with a reported high concordance to manual scoring in multiple paradigms (Tatem et al., 2014). To further ensure accuracy, we manually checked approximately 20% of the sessions to verify that the automated scoring matched the animal's actual position and movements. No discrepancies were detected, supporting the reliability of the software's measures in our setup.

### Statistical analysis

The time spent in the center of the open field, center entries, total distance traveled, and the time spent in the light compartment of the light/dark box and IHC staining were analyzed using a generalized estimating equation (GEE) model, a multivariable, repeated-measures, regression model. This approach allows for modeling data with multiple measures per animal and adjusts for potential confounding variables like sex and litter. Sex, genotype, and group were used as factors. Sex and litter were not significant and were therefore removed from the final analyses. Nonsignificance is defined as *p* > 0.05; *p*-values for all significant results are reported in the Results section.

To ensure that our study was sufficiently powered to detect group differences, we conducted a post hoc power analysis for each GEE model using G*Power (Faul et al., 2007). Effect sizes (Cohen's *f*^2^) were derived from the Wald chi-square (*χ*^2^) values reported in the GEE output using the formula *f*^2 ^=  *χ*^2^ / *N*, where *N* represents the total number of animals analyzed in each model.

For the WT/KO dataset (*N* = 44), the Wald chi-square was *χ*^2^ = 17.6 (df = 5, *p* < 0.001), yielding an effect size of *f*^2^ = 0.40, which resulted in 87.2% power at *α* = 0.05. For the KI dataset (*N* = 24), the Wald chi-square was *χ*^2^ = 13.7 (df = 3, *p* < 0.001), yielding an effect size of *f*^2^ = 0.57, achieving 81.3% power at *α* = 0.05.

## Results

### Seizure latency and duration are unaffected by treatment or MC4R knock-out

**Figure 1.**
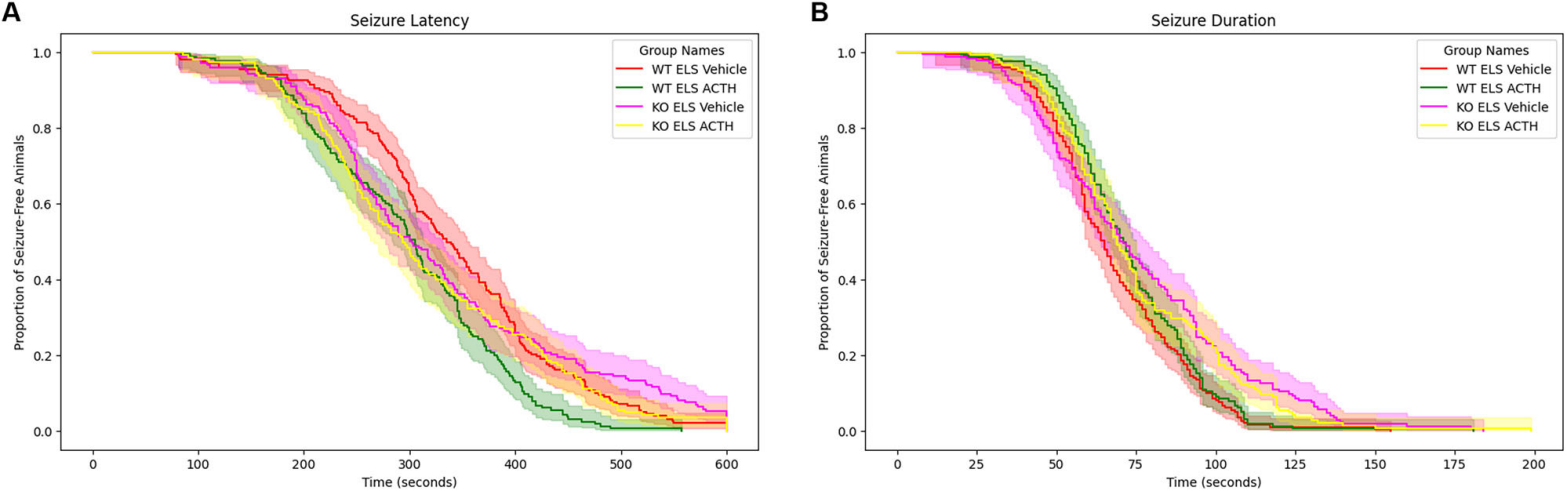
Seizure parameters are not altered by ACTH treatments. Treatment with ACTH does not alter latency to flurothyl seizure compared with vehicle-treatment groups across the different genotypes (***A***). Similarly, treatment with ACTH did not significantly alter seizure duration across different genotypes (***B***).

We first asked whether our treatment differentially affected seizure induction in any of our groups. To do this, we analyzed seizure latency and duration. The mean latency and duration of seizures, along with their standard errors of the mean (SEM), were calculated for each group: WT ELS vehicle (latency, 344.73 ± 7.62 s; duration, 68.06 ± 1.57 s), WT ELS ACTH (latency, 298.52 ± 7.06 s; duration, 72.97 ± 1.66 s), KO ELS vehicle (latency, 326.77 ± 9.59 s; duration, 80.88 ± 3.47 s), and KO ELS ACTH (latency, 314.71 ± 9.69 s; duration, 75.55 ± 2.31 s). The Cox proportional hazards analysis was employed to assess the impact of treatment and genotype on seizure latency and duration, adjusting for multiple measurements per animal and day-specific effects. For seizure latency, the analysis revealed no significant effect of treatment (*p* = 0.73, Cox proportional hazards) or genotype (*p* = 0.81, Cox proportional hazards) across days (Fig. 1*A*). For seizure duration, the analysis showed no significant effect of treatment (*p* = 0.16, Cox proportional hazards) or genotype (*p* = 0.22, Cox proportional hazards) across days (Fig. 1*B*). Overall, our findings suggest that neither treatment nor genotype significantly influenced seizure latency or duration when adjusted for day-specific effects and multiple measurements per animal.

### ELS did not affect exploration and spontaneous activity in open field task

**Figure 2.**
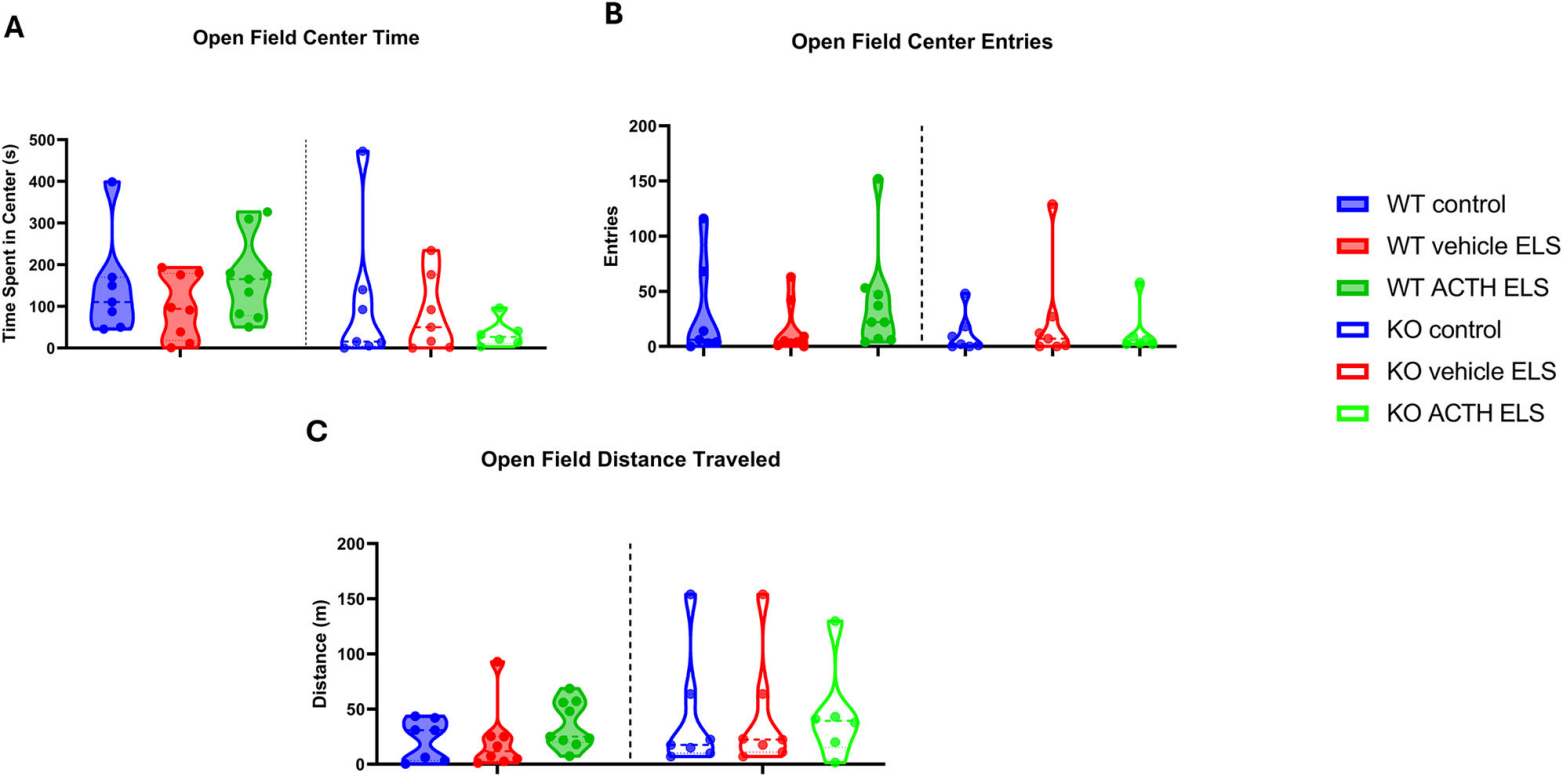
No behavioral differences in open field task parameters. There were no significant differences in the time spent in the center of the open field between the WT and KO groups (***A***). Similarly, the number of entries into the center did not differ significantly between the WT and KO groups (***B***). The total distance traveled by the subjects also showed no significant variation across the WT and KO groups (***C***).

The open field behavior test was conducted to assess spontaneous activity and exploration behavior in the experimental groups. This task measures the time spent in the center of the open field, the number of entries into the center, and the total distance traveled. These parameters provide insights into the animals' willingness to explore a new environment and their activity. There were no significant differences in the time spent in the center of the open field between the control, vehicle-treated ELS, and ACTH-treated ELS (*p* > 0.05, GEE; Fig. 2*A*). Similarly, the number of entries into the center did not differ significantly between the groups (*p* > 0.05, GEE; Fig. 2*B*). Lastly, there was no significant variation in the total distance traveled by the animals across the different groups (*p* > 0.05, GEE; Fig. 2*C*). These results indicate that neither treatment nor genotype had a significant effect on spontaneous activity or exploration in the open field task (genotype–treatment effect, *p* > 0.05, GEE).

### ACTH ameliorates anxiety in light/dark box task

The light/dark box task was conducted to specifically assess anxiety-related behavior in the experimental groups. This task measures the time spent in the light zone of a box with both lighted and dark chambers, based on the natural aversion of rodents to brightly lit areas. Increased time in the light zone indicates reduced anxiety. Unlike the open field task, which primarily measures general locomotor activity and exploration, the light/dark box task is more sensitive to detecting anxiety-related behaviors.

**Figure 3.**
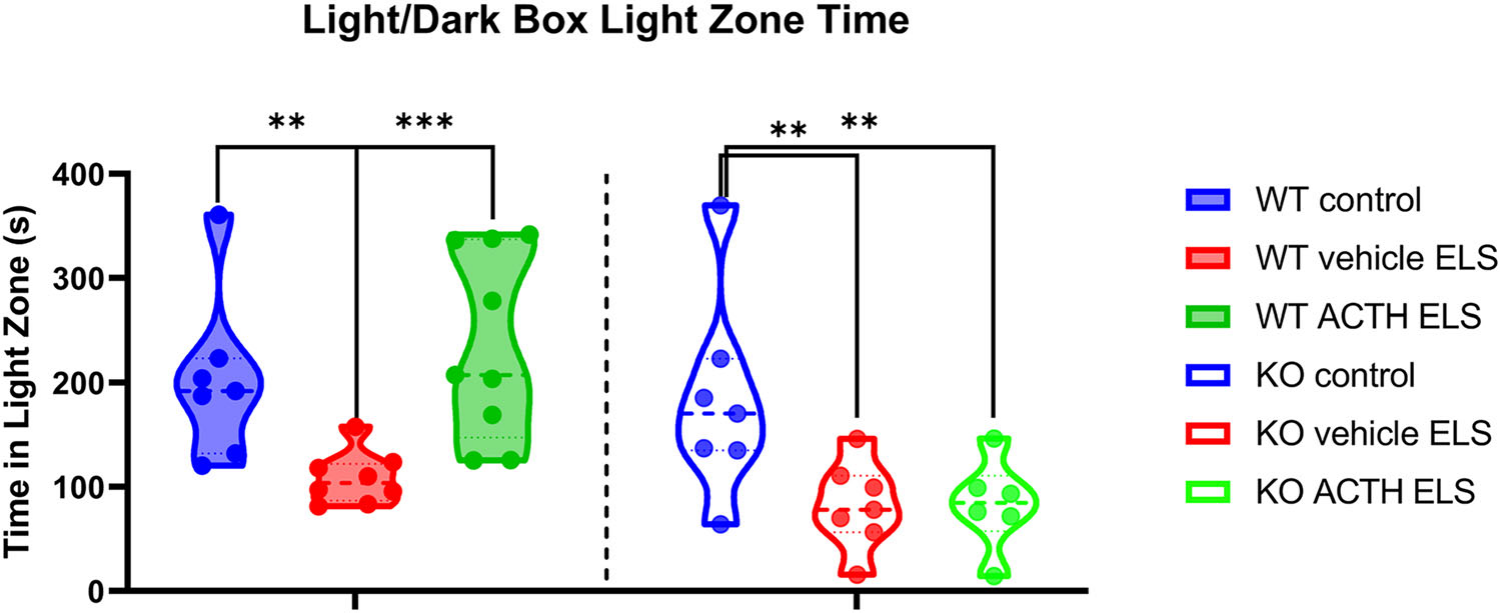
ACTH ameliorates the anxiety phenotype in mice with ELS. The light/dark box task was used to assess anxiety-related behavior by measuring the time spent in the light zone and the number of entries into the light zone. WT ELS mice treated with ACTH spent significantly more time in the light zone compared with WT ELS vehicle-treated mice. ACTH did not rescue the anxiety phenotype in MC4R KO ELS ACTH-treated mice compared with MC4R KO ELS vehicle-treated mice.

WT vehicle-treated ELS mice spent significantly less time in the light zone compared with the WT controls (Fig. 3; *p* = 0.005, GEE). Interestingly, ACTH was able to ameliorate the anxiety in mice with a history of ELS. ACTH-treated ELS mice spent significantly more time in the light zone compared with the vehicle-treated group (*p* = 0.00049, GEE). Similarly to the WT group, the MC4R KO vehicle-treated ELS spent significantly less time in the light zone compared with MC4R KO controls (*p* = 0.003, GEE); however, ACTH treatment was not able to ameliorate the anxiety (*p* = 0.005 to the KO control group) indicating a MC4R-dependent mechanism for this amelioration, with a significant genotype–treatment effect (*p* = 0.000061, GEE).

### Acute astrocyte dysfunction in the prefrontal cortex and hippocampus after recurrent early-life seizures

**Figure 4.**
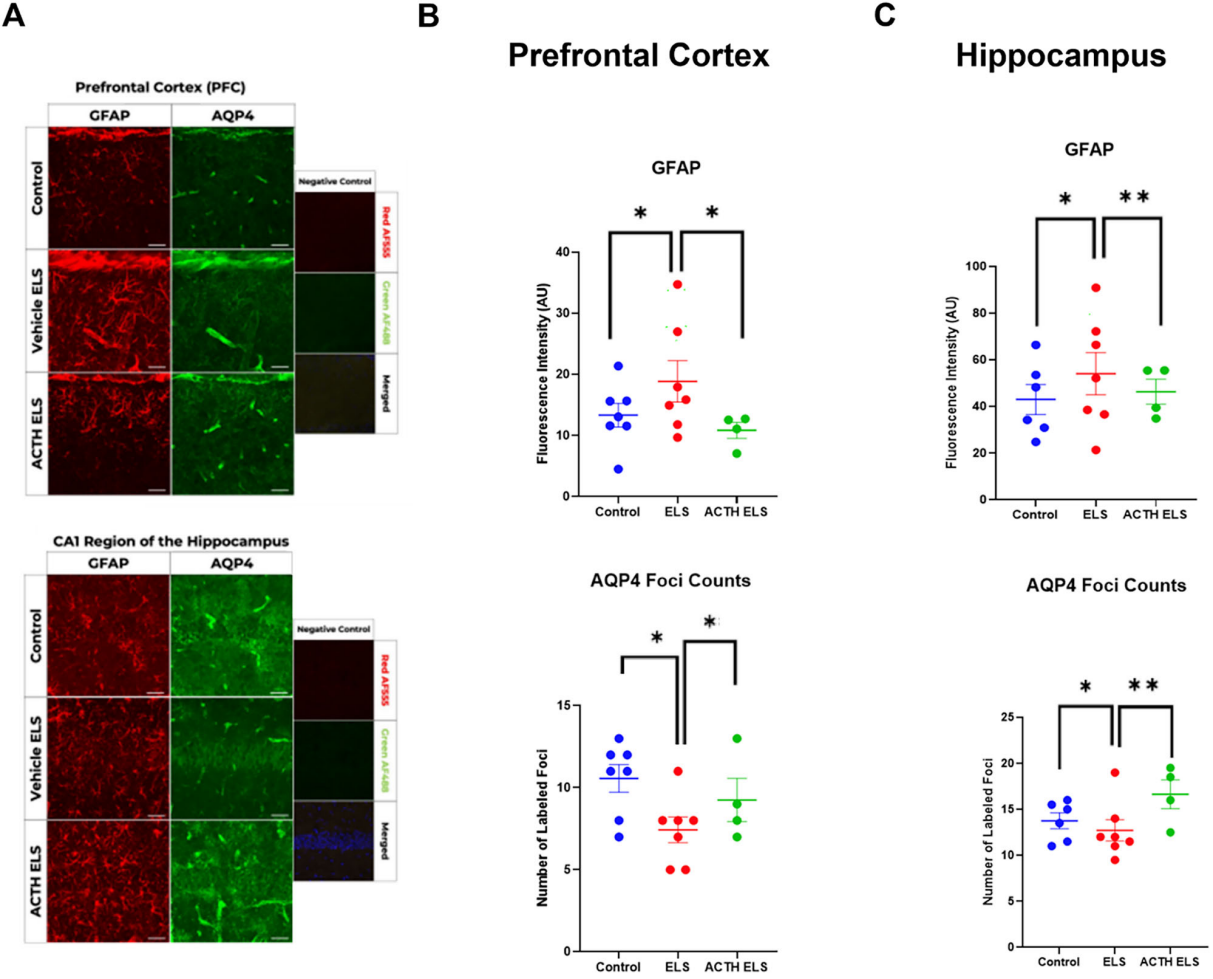
ACTH mitigates the astrocytic dysfunction in mice with ELS. Immunohistochemistry assessed astrocytic dysfunction after early-life seizures (***A***). Mice treated with ACTH showed a recovery in astrocytic function. ACTH normalized GFAP and AQP4 levels in the PFC after early-life seizures (***B***, ***C***). ACTH normalized GFAP and AQP4 levels in the HC after early-life seizures (***B***, ***C***).

Extensive research has focused on neuronal function, neuronal alterations, and their long-term consequences following early-life seizures. For instance, it was shown that early-life seizures can disrupt the hippocampal–prefrontal cortex network and lead to cognitive deficits and psychiatric-like manifestations (Ruggiero et al., 2024). It was further shown that these deficits and manifestations occur due to changes in synaptic plasticity, such as an aberrant increase in long-term potentiation (LTP), rather than neuronal loss (Ruggiero et al., 2024). Other studies have also demonstrated that ELS does not result in cell death; however, it causes alterations in short- and long-term plasticity (Karnam et al., 2009; Hernan et al., 2013) and reduced neurogenesis (Holmes et al., 1998, 1999, 2015; De Rogalski Landrot et al., 2001; McCabe et al., 2001). In addition to that, after ELS, there is a decrease in inhibitory currents in the hippocampus and neocortex and hyperexcitation in the neocortex (Isaeva et al., 2006, 2010). While much is known about the neuronal alterations following ELS, there remains a significant gap in our understanding of astrocytic responses, their contributions to early-life epilepsy and seizures, and their modulation of the MC4R effect. To address this gap, we used immunohistochemistry to examine acute astrocyte protein expression in the prefrontal cortex and hippocampus after ELS with and without ACTH treatment as astrocytes are involved in various neuroprotective and neuroinflammatory processes that could influence seizure outcomes (Fig. 4*A*). Forty-eight hours after seizures, we saw a significant increase in GFAP expression in both brain regions (*p* = 0.048, GEE; Fig. 4*B*,*C*), indicating enhanced astrocytic activation and gliosis. We also saw a significant decrease in AQP4 expression in both brain regions after ELS (*p* = 0.003, GEE; Fig. 4*B*,*C*). Dysregulation of AQP4 has been linked to increased seizure susceptibility through astrocyte proliferation, hypertrophy, impaired water balance, and edema (Heuser et al., 2010; Hubbard et al., 2016; Szu et al., 2020; Lu et al., 2021; Vezzani et al., 2022; Sumadewi et al., 2024). The results suggest that ELS is associated with astrocytic dysfunction. ACTH treatment was able to recover GFAP and AQP4 levels. These findings highlight the acute impact of ELS on astrocytic populations and the potential of ACTH treatment to mitigate these effects.

### Cell-type–specific reexpression of MC4R in neurons and astrocytes

Given that astrocyte protein expression was altered after ELS and that MC4Rs are expressed in both neurons and astrocytes, we asked whether MC4Rs in these cell types differentially contribute to anxiety prevention following ELS. We therefore generated knock-in (KI) mice by re-expressing MC4R in either neurons or astrocytes, followed by ELS and ACTH treatment. We emphasize that the GFAP-Cre line used here has been shown to colocalize specifically with GFAP+ astrocytes in multiple brain regions (Jia et al., 2020, 2021; Itoh et al., 2023), and the Syn1-Cre line exhibits robust Cre expression in neurons (Zhu et al., 2001; Zheng et al., 2013). Although no Cre driver is 100% cell-type restricted, these lines are widely regarded as reliable tools to target neurons or astrocytes, respectively, with extensive published evidence, particularly lineage-tracing and immunohistochemical analyses, demonstrating minimal off-target expression (Luo et al., 2020). By using these well-characterized lines, we minimized potential confounds arising from ectopic recombination and effectively isolated the roles of neuronal versus astrocytic MC4R in our ELS model.

**Figure 5.**
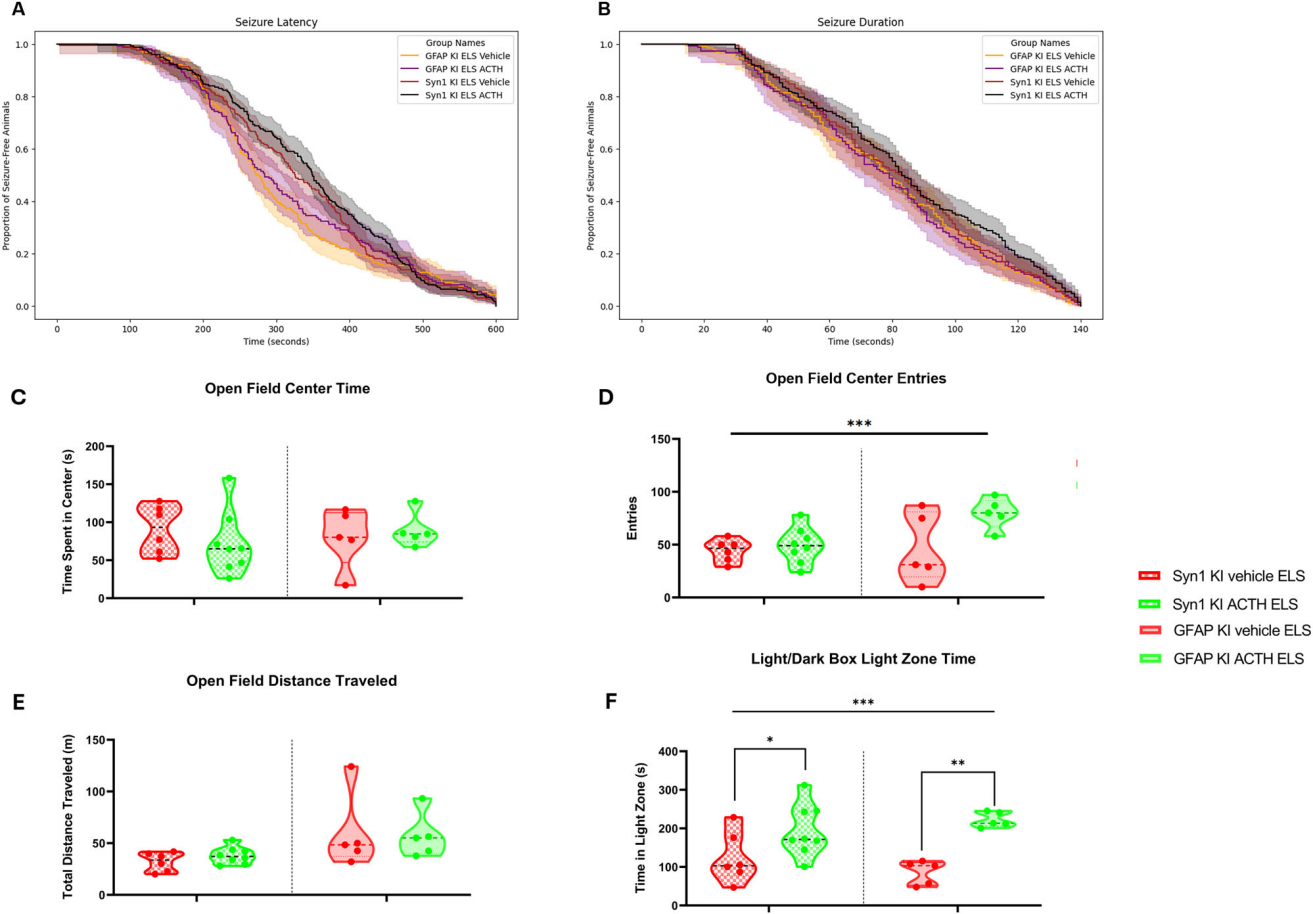
ACTH-treated KI mice with ELS show less anxiety with no differences in seizure parameters or spontaneous activity. Survival analysis plot indicates no differences in latency to tonic–clonic seizure between animals with MC4R reexpression in astrocytes and without treatment with ACTH (yellow vs purple line and envelope) or animals with MC4R reexpression in neurons with and without treatment with ACTH (red vs blue line; ***A***). Similarly, treatment with ACTH did not significantly alter seizure duration in different groups of KI mice (***B***). There were no significant differences in the time spent in the center of the open field between the vehicle-treated and ACTH-treated KI ELS groups (***C***). Similarly, the number of entries into the center did not differ significantly between the vehicle-treated and ACTH-treated KI ELS groups; however, there is a genotype–treatment effect with post hoc comparisons showing that treatment with ACTH in MC4R astrocyte KI animals was significantly associated with an increase in the number of center entries compared with other groups (***D***). The total distance traveled by the subjects also showed no significant variation across the vehicle-treated and ACTH-treated KI ELS groups (***E***). Both KI ELS mice treated with ACTH spent significantly more time in the light zone compared with KI ELS vehicle-treated mice; however, there is a genotype–treatment effect with post hoc comparisons showing that treatment with ACTH in MC4R astrocyte KI animals was significantly associated with an increase in the time spent in the light zone compared with other groups (***F***).

KI of the receptor did not change seizure parameters, and there were no significant effects of treatment (*p* = 0.68 for latency and *p* = 0.40 for duration, GEE) on either the astrocyte or neuron reexpression group (Fig. 5*A*,*B*; GFAP KI ELS vehicle latency 309.29 ± 9.28 s, duration 79.02 ± 2.33 s; GFAP KI ELS ACTH latency 317.83 ± 10.86 s, duration 78.50 ± 2.64 s; Syn1 KI ELS vehicle latency 332.98 ± 8.89 s, duration 82.04 ± 2.21 s; and Syn1 KI ELS ACTH latency 347.32 ± 8.04 s, duration 85.19 ± 2.14 s).

We saw no significant differences in time spent in the center (Fig. 5*C*), number of entries to the center (Fig. 5*D*), or the total distance traveled (Fig. 5*E*) between the vehicle-treated and ACTH-treated ELS knock-in (KI) groups in the open field. It is important to note that the vehicle-treated groups represent two distinct Cre+ genotypes: Syn1-Cre+ for neuronal MC4R re-expression and GFAP-Cre+ for astrocytic MC4R re-expression. Although both are vehicle-treated, they differ in the promoter driving Cre (Syn1 vs GFAP), which can possibly introduce baseline behavioral differences. However, our analysis uses genotype and treatment as factors to account for these baseline differences with no significant difference between the vehicle-treated ELS KI groups for center entries (GEE, *p* = 0.758634), distance (GEE, *p* = 0.063050), or time in light zone (GEE, *p* = 0.727150). A significant genotype–treatment effect (*p* = 0.000006, GEE) for open field center entries indicates that the treatment differentially affected the two reexpression groups. Post hoc comparisons show that treatment with ACTH in animals where MC4Rs were reexpressed in astrocytes was significantly associated with an increase in the number of center entries compared with MC4R neuron KI groups (*p* = 0.00000374 to the syn1-cre ELS vehicle group and *p* = 0.000154 to the syn1-cre ELS ACTH group).

In the light/dark box task, ACTH treatment decreased anxiety in both the animals with MC4R reexpressed in neurons and animals with the receptor reexpressed in astrocytes (Fig. 5*F*). Neuronal KI ELS mice treated with ACTH showed a significant increase in light zone time compared with neuronal KI mice treated with vehicle (*p* = 0.034, GEE). Similarly, the astrocyte KI ACTH-treated mice spent more time in the light zone compared with the vehicle-treated astrocyte KI ELS mice (*p* = 0.006, GEE). Notably, there is a genotype–treatment effect (*p* = 0.00089, GEE) for the light zone time. This was again driven by the ACTH-treated animals in the astrocyte reexpression group, whose time spent in the light zone was significantly higher than all other groups (*p* < 0.001, GEE). This suggests that reexpression in astrocytes was more effective at recovering the treatment effect than reexpression in neurons.

## Discussion

The present study aimed to elucidate the mechanism by which ACTH can improve psychiatric comorbidities after ELS. Deficits are a well-documented consequence of ELS, with up to 65% of patients experiencing impairments in anxiety, learning, memory, and executive function (Lodhi and Agrawal, 2012; Reilly et al., 2014; Ahmed et al., 2022; Jensen-Willett et al., 2022; Singh et al., 2022; Sayed et al., 2023). These deficits are particularly concerning as they can persist into adulthood, significantly impacting quality of life (Lodhi and Agrawal, 2012; Reilly et al., 2014; Ahmed et al., 2022; Sayed et al., 2023). Traditional antiseizure medications primarily focus on controlling seizure activity but do not address these comorbidities associated with epilepsy (Lodhi and Agrawal, 2012; Valeta, 2017; Khalife et al., 2022). This gap in treatment highlights the need for therapeutic strategies that can mitigate these comorbidities. Our study investigated ACTH's effect on anxiety, showing MC4R-dependent improvements, which challenge the notion that ACTH's primary effects are mediated through MC2Rs in the adrenal cortex for systemic glucocorticoid release (Roy et al., 2007; Novoselova et al., 2019). In support of actions above and beyond corticosteroids, we previously showed that dexamethasone treatment failed to recapitulate the positive effects on behavior seen with ACTH treatment (Massey et al., 2016) and that ACTH, but not dexamethasone, normalizes gene expression after ELS with no effects of ACTH on seizure parameters (Massey et al., 2016; Brabec et al., 2022).

ACTH belongs to the melanocortin family of neuropeptide hormones, all derived from proopiomelanocortin (POMC), and binds with varying affinity to melanocortin receptors (MCRs). MC2R is exclusively activated by ACTH, while the other four receptors respond to both ACTH and other POMC peptides, with different binding affinities. These G-protein-coupled receptors also exhibit distinct tissue distributions and functions (Mountjoy et al., 1992; Wikberg, 1999; Yang, 2011; Cai and Hruby, 2016; Massey et al., 2016). In the brain, melanocortin receptor MC4R was shown to have important roles in the pathophysiology of various neurological disorders like Alzheimer's disease and cerebral ischemia. Activation of MC4R has been shown to promote neuroprotection and enhance cognitive function through multiple mechanisms. It was previously demonstrated that melanocortins protect against the progression of Alzheimer's disease in triple-transgenic mice (3xTg); the study found that treatment with α-melanocyte-stimulating hormone (NDP-α-MSH) reduced cerebral cortex and hippocampus phosphorylation levels of amyloid/tau cascade proteins, inflammation, and apoptosis. Treated mice demonstrated decreased neuronal loss and improved learning and memory (Giuliani et al., 2014). Another study, utilizing the TgCRND8 Alzheimer's disease mouse model, found that α-MSH treatment prevents GABAergic neuronal loss and improves cognitive function. Treated mice exhibited improved spatial memory and reduced anxiety during the Y-maze task (Ma and McLaurin, 2014). In the context of cerebral ischemia, MC4R agonists counteract late inflammatory and apoptotic responses postischemia in a transient global brain ischemia model. Treatment with MC4R agonists reduced the levels of proinflammatory cytokines and apoptosis markers, leading to enhanced neuronal survival and functionality further improving cognitive performance in the Morris water maze task (Spaccapelo et al., 2011).

Furthermore, other studies demonstrated that activating MC4R has an impact on neuronal viability and synaptic plasticity. α-MSH treatment was shown to rescue neurons from excitotoxic cell death following kainic acid-induced damage resulting in a significantly higher number of viable neurons in the hippocampal CA1 pyramidal cell layer (Forslin Aronsson et al., 2007). Moreover, activation of MC4R enhanced synaptic plasticity by increasing the number of mature dendritic spines and enhancing the surface expression of AMPA receptor subunit GluA1 mediating neurotransmission enhancement and hippocampal long-term potentiation (Shen et al., 2013).

In our study, ACTH treatment administered 1 h before each seizure day did not alter seizure latency or duration, which shows that ACTH's therapeutic effects extend beyond seizure modification and are likely mediated by MC4R pathways unrelated to seizure modulation (Hernan et al., 2014; Massey et al., 2016). This finding challenges the prevailing assumption that deficits after ELS are a result of the seizures themselves.

The open field task results indicated that ELS did not significantly affect general locomotor activity or exploration behavior, as there were no differences in time spent in the center, number of entries into the center, or total distance traveled across the groups. This suggests that ELS and subsequent ACTH treatment do not impact the animals' general activity levels or willingness to explore a new environment. In contrast, the light/dark box task revealed significant anxiety-related behaviors in ELS mice, which were ameliorated by ACTH treatment. WT ELS mice exhibited increased anxiety, spending less time in the light zone compared with controls. ACTH treatment significantly reduced this anxiety-like behavior, indicating its potential therapeutic effect. However, in MC4R KO mice, ACTH treatment did not ameliorate anxiety, showing that the anxiolytic effects of ACTH are mediated through MC4R signaling pathways.

Immunohistochemistry staining was performed for important astrocytic proteins like GFAP and AQP4. The selected proteins play an essential role in astrocytic function and were shown to be altered in epilepsy. Astrocytes, glial cells in the brain, play a critical role in supporting neuronal function, modulating synaptic transmission, and maintaining homeostasis within the central nervous system (Vasile et al., 2017; Siracusa et al., 2019; Vezzani et al., 2022). In epilepsy, astrocytes undergo significant alterations affecting astrocyte channels, transporters, and metabolism which are directly linked to epileptogenesis (Binder and Steinhäuser, 2021; Chen et al., 2021; Çarçak et al., 2023). Specifically, disruptions in potassium, and water homeostasis, contribute to the seizure predisposition, epilepsy onset, and hyperexcitability characteristic of epilepsy (Kang et al., 2005; Tian et al., 2005; Binder and Steinhäuser, 2021). Furthermore, astrocytes' role in synchronizing neuronal activity and regulating synaptic transmission and plasticity, by releasing transmitters and maintaining calcium signaling, makes them essential for cognitive and psychiatric functions like learning, memory, and stress responses (Koyama, 2015; Vasile et al., 2017; Siracusa et al., 2019; Kaul et al., 2022; Escalada et al., 2024). Disruptions in astrocyte function have also been linked to mood and cognitive disturbances, suggesting that interventions restoring astrocyte homeostasis could alleviate diverse neuropsychiatric symptoms (Koyama, 2015; Adamsky et al., 2018; Kaul et al., 2022). Prolonged stimulation of hippocampal astrocytes, for instance, has been found to impair spatial memory and working memory, indicating that astrocytic reactivity can negatively impact cognitive function (Bohmbach and Henneberger, 2024; Kim et al., 2024). However, sufficient activation of hippocampal astrocytes led to enhanced calcium activity sufficient to induce long-term potentiation (LTP) which is a cellular correlate of learning and memory. Mice performing contextual fear conditioning and T-maze tasks and undergoing hippocampal astrocyte activation during the acquisition phase demonstrated an improved memory recall in both tasks the following day (Adamsky et al., 2018). Furthermore, another study showed that inhibition of activated astrocytes after inflammation was able to ameliorate anxiety and depressive-like behaviors induced by lipopolysaccharide in addition to decreasing GFAP and increasing BDNF levels in the brain (Wang et al., 2019). These studies highlight the importance of astrocyte homeostasis and the important role of astrocytes in neuropsychiatric deficits.

Increased levels of GFAP are observed in epilepsy and are indicative of astrogliosis (Vezzani et al., 2022; Sumadewi et al., 2024). AQP4 is a water channel protein involved in water homeostasis and balancing potassium concentration, and its altered expression can lead to disrupted water regulation which is linked to increased worsened seizure activity (Szu et al., 2020; Lu et al., 2021). This shows the importance of these proteins for proper astrocytic function.

Our immunohistochemistry staining analysis demonstrated significant astrocytic dysfunction in both the prefrontal cortex and hippocampus following ELS, as evidenced by increased GFAP levels and decreased AQP4 expression 48 h after the last seizure. While direct evidence linking MC4R activation to changes in GFAP or AQP4 in epilepsy models is scarce, melanocortin signaling, including MC4R activation, exerts anti-inflammatory and astrocytic modulation in CNS disease (Caruso et al., 2007; Chen et al., 2021) opening an important question about the effect of MC4R activation on astrocytic protein expression normalization. GFAP upregulation and AQP4 dysregulation are hallmark features of astrocyte reactivity that can exacerbate seizure susceptibility and neural dysfunction. Thus, it is plausible that MC4R-dependent astrocyte modulation underlies the normalization of GFAP and AQP4 we observed (Liu et al., 2015). First, MC4R is G-coupled, elevating intracellular cAMP upon activation, which can regulate downstream signaling cascades that modulate glial inflammation and gliosis (Caruso et al., 2007; Paco et al., 2016; Zhou et al., 2019; Chen et al., 2021). Second, changes in cAMP-dependent pathways have been shown to affect astrocytic hypertrophy and the expression of intermediate filaments, including GFAP (Freeman et al., 1989; Segovia et al., 1994; Cebolla et al., 2008; Yang and Wang, 2015). Third, water channel proteins like AQP4 can be influenced by signaling events that also involve cAMP-regulated transcription factors (Song and Gunnarson, 2012; Kitchen et al., 2020). Indeed, ACTH treatment effectively normalized both markers, suggesting its potential to mitigate acute astrocytic dysfunction induced by ELS. The recovery of these astrocytic markers highlights the therapeutic potential of ACTH in restoring normal astrocytic function and, by extension, improving neural homeostasis.

The changes in astrocyte protein expression after ELS and the ability of ACTH to improve chronic anxiety in WT mice, but not in KO mice, raised a question about the impact of neuronal and glial MC4R expression on ACTH’s positive effects. To address the question of cell-specific expression of MC4R, we reexpressed the MC4R selectively into neuronal populations or astrocytic populations. We observed that KI mice did not show significant differences in open field parameters. However, the significant genotype–treatment effect for the number of entries, with the GFAP KI–ACTH treatment showing a significant effect compared with the groups with MC4R neuron reexpression, highlights that the genotype influences the ACTH's effect on exploration and activity levels in the open field. In the light/dark box task, anxiety-like behaviors decreased in both Syn1 KI and GFAP KI mice treated with ACTH. Notably, the significant genotype–treatment effect for the light zone time, with the GFAP KI–ACTH treatment showing a significant effect compared with all other groups, suggests that the effect of treatment on reducing anxiety was more pronounced in the GFAP KI genotype group. This supports the role of MC4R in both neuronal and astrocytic populations in modulating anxiety responses following ELS; however, the expression of MC4R in astrocytes appears to have a greater impact, with less anxiety, after ELS compared with its expression in neurons.

Our findings highlight the complex interplay between ELS, astrocytic function, and behavioral outcomes. ACTH treatment, while not influencing seizure parameters directly, shows promise in reducing ELS-associated anxiety and astrocytic dysfunction. Future research should explore precise mechanisms underlying ACTH's therapeutic effects.

## Synthesis

Reviewing Editor: Viji Santhakumar, University of California Riverside Decisions are customarily a result of the Reviewing Editor and the peer reviewers coming together and discussing their recommendations until a consensus is reached. When revisions are invited, a fact-based synthesis statement explaining their decision and outlining what is needed to prepare a revision will be listed below. The following reviewer(s) agreed to reveal their identity: Jamie Maguire. Note: If this manuscript was transferred from JNeurosci and a decision was made to accept the manuscript without peer review, a brief statement to this effect will instead be what is listed below.

The manuscript investigates the ability of ACTH to influence anxiety-like behaviors associated with early life seizures. This is an important and underexplored area of research. The current manuscript demonstrates that ACTH is capable of reducing anxiety-like behaviors associated with early life seizures, through a mechanism involving Melanocortin 4 Receptors (MC4R) on both astrocytes and neurons. This research is a nice extension of previous work, elucidating a novel mechanism mediating the therapeutic effects of ACTH. While the study is narrow in scope, it is experimentally sound and informative to the field.

1. The n numbers are low for behavioral experiments. Providing a power analysis may help alleviate concerns that these experiments are underpowered. Please reassess statistical analysis in figure 4c

2. The link to GFAP and AQP4 is still weak. The discussion of GFAP and AQP4 (starting at Line 437) would benefit from discussing a link to MC4R.

3. The research is framed around the impact of ACTH on cognitive outcomes, but cognitive outcomes are not tested here. This framing is likely based on previous work by this group. It would be more beneficial to frame the story around anxiety given the focus of the current study. For instance, the first sentence in the Discussion states "The present study aimed to elucidate the mechanism by which ACTH can improve cognitive outcome after ELS." Similarly, line 385 indicates "cognitive comorbidities" where psychiatric comorbidities would be more appropriate.

4. Fig 2: Appears to have high thigmotaxis behavior, explain how these animals were handled, how many people handled them, where there people in the room when experiment began? Overlay individual data points.

5. Additional information of software validation needs to be provided.

6. Control experiments are needed to demonstrate that knock in affected just the intended cell type and nowhere else

7. Please indicate if animals littermates? With respect to Fig. 3, how were the conditions between cages kept constant so as to not affect their anxiety? Overlay individual data points.

8. Fig 5: Shouldn't the vehicle treated controls be the same whether its neurons or astrocytes? why are there differences between controls specifically in D and E? Why does promoter cause this difference? overlay individual data points.
